# Immediate percutaneous coronary intervention in NSTE-ACS: the sun is not hurried by early risers

**DOI:** 10.1007/s12471-016-0806-x

**Published:** 2016-02-09

**Authors:** P. Knaapen

**Affiliations:** Department of Cardiology, VU University Medical Center, Amsterdam, The Netherlands

Primary percutaneous coronary intervention (PCI) has unequivocally proven to be the optimal reperfusion treatment strategy in patients with ST-segment elevation myocardial infarction, whereby timing is simply an issue of the sooner the better. In recent years, it has additionally become apparent that an early invasive approach is also beneficial in patients with non-ST-segment elevation acute coronary syndromes (NSTE-ACS). The definition of early in this subset of patients is, however, ambiguous. Based on risk stratification parameters, high-risk patients (i.e. GRACE score > 140) are preferably subjected to invasive diagnosis and potential PCI within 24 h whereas in low-intermediate risk patients, urgency of coronary angiography is of lesser importance and advocated to occur within a timeframe of 72 h after the initial diagnosis. These guidelines dictate the timeframe in which the invasive approach should be employed at the latest but do not indicate whether an immediate treatment may yield an even better outcome [1].

In this issue of *the Netherlands Heart Journal*, Oosterwerff et al. report on the long-term follow-up data of the OPTIMA trial exploring the effects of an immediate vs. deferred PCI in NSTE-ACS patients with coronary anatomy amenable for percutaneous intervention [2]. Even though underpowered to draw definite conclusions, the results are intriguing. After 5 years, there was no difference in the combined endpoint but late myocardial infarction (> 30 days after randomisation) was observed significantly more frequently in the immediate treatment arm and was linked to the index vessel in over half of the cases. The original results from the OPTIMA trial likewise suggested that an immediate invasive treatment of NSTE-ACS patients is associated with a poorer 30-day outcome and conveys an increased rate of peri-procedural myocardial infarction [3]. Whereas the potential early detrimental effects of immediate intervention are likely to be attributed to increased thrombus load with inherent phenomena such as no-reflow and thrombotic side branch occlusion provoked by the procedure, it is less clear how long-term prognosis 30 days after hospitalisation could be related to the timing of the index procedure. Postulated mechanisms include the fact that deferred intervention may result in restoration of vascular tone and thus may facilitate more optimal sizing of stents potentially preventing late stent thrombosis and restenosis. On the other hand, it should be acknowledged that the study is hampered by uneven distribution of baseline patient characteristics. The immediate treatment arm comprised significantly more patients who had undergone previous bypass surgery indicating a more advanced stage of coronary artery disease. In fact, half of the late myocardial infarctions in the immediate arm occurred in non-index vessels with no apparent relation to the initial procedure. Furthermore, the current data are not in line with previous studies such as the ABOARD trial, which failed to display any differences in outcome of immediate vs. deferred intervention to the next working day [4].

The debate pertaining to the optimal timing of intervention in patients presenting with NSTE-ACS will therefore continue as results from ongoing trials as the OPTIMA 2 and TRANSIENT are eagerly awaited [5]. In the meantime, it is reassuring to realise that acute interventions in these patients are not mandatory and medical stabilisation with antithrombotic agents appears to be non-inferior and may even be associated with improved short- and long-term outcome. This is particularly convenient from a logistical point of view as it avoids the necessity for patients to be rushed to centres with 24/7 PCI coverage and they can safely be hospitalised at local community hospitals for initial medical treatment prior to coronary angiography in the ensuing days [6]. This prevents a substantial additional burden on catheterisation laboratory facilities, which was one of the main reasons for the slow inclusion rate of the OPTIMA trial and ultimately resulted in termination of the trial before the required number of patients was enrolled [3]. Another pivotal issue that arises is whether coronary angiography should be restricted to PCI centres in these patients. The OPTIMA trial revealed that 57 % of patients displayed coronary pathology amenable for ad hoc PCI. Mere diagnostic coronary angiography without the ability to perform intervention in NSTE-ACS therefore results in unnecessary secondary procedures and treatment delay in the majority of patients. These arguments could act as a plea to transfer all NSTE-ACS patients within 24 or 72 h, depending on the GRACE risk score, to interventional clinics in order to offer appropriate treatment in a timely fashion. This will require even closer collaboration between PCI centres and their satellite partners to further optimise patient care. A goal, nonetheless, that is worth pursuing.

## Funding

None.
